# Novel microarchitecture of human endometrial glands: implications in endometrial regeneration and pathologies

**DOI:** 10.1093/humupd/dmab039

**Published:** 2021-12-07

**Authors:** Nicola Tempest, Christopher J Hill, Alison Maclean, Kathleen Marston, Simon G Powell, Hannan Al-Lamee, Dharani K Hapangama

**Affiliations:** 1 Department of Women’s and Children’s Health, Centre for Women’s Health Research, Institute of Life Course and Medical Sciences, University of Liverpool, Member of Liverpool Health Partners, Liverpool, UK; 2 Liverpool Women's NHS Foundation Trust, Member of Liverpool Health Partners, Liverpool, UK; 3 Hewitt Centre for Reproductive Medicine, Liverpool Women’s NHS Foundation Trust, Liverpool, UK

**Keywords:** endometrium, 3D microarchitecture, regeneration, endometriosis, adenomyosis, endometrial stem progenitor cells, endometrial polyps, placenta accreta spectrum, fertility

## Abstract

**BACKGROUND:**

Human endometrium remains a poorly understood tissue of the female reproductive tract. The superficial endometrial functionalis, the site of embryo implantation, is repeatedly shed with menstruation, and the stem cell-rich deeper basalis is postulated to be responsible for the regeneration of the functionalis. Two recent manuscripts have demonstrated the 3D architecture of endometrial glands. These manuscripts have challenged and replaced the prevailing concept that these glands end in blind pouches in the basalis layer that contain stem cells in crypts, as in the intestinal mucosa, providing a new paradigm for endometrial glandular anatomy. This necessitates re-evaluation of the available evidence on human endometrial regeneration in both health and disease in the context of this previously unknown endometrial glandular arrangement.

**OBJECTIVE AND RATIONALE:**

The aim of this review is to determine if the recently discovered glandular arrangement provides plausible explanations for previously unanswered questions related to human endometrial biology. Specifically, it will focus on re-appraising the theories related to endometrial regeneration, location of stem/progenitor cells and endometrial pathologies in the context of this recently unravelled endometrial glandular organization.

**SEARCH METHODS:**

An extensive literature search was conducted from inception to April 2021 using multiple databases, including PubMed/Web of Science/EMBASE/Scopus, to select studies using keywords applied to endometrial glandular anatomy and regeneration, and the references included in selected publications were also screened. All relevant publications were included.

**OUTCOMES:**

The human endometrial glands have a unique and complex architecture; branched basalis glands proceed in a horizontal course adjacent to the myometrium, as opposed to the non-branching, vertically coiled functionalis glands, which run parallel to each other as is observed in intestinal crypts. This complex network of mycelium-like, interconnected basalis glands is demonstrated to contain endometrial epithelial stem cells giving rise to single, non-branching functionalis glands. Several previous studies that have tried to confirm the existence of epithelial stem cells have used methodologies that prevent sampling of the stem cell-rich basalis. More recent findings have provided insight into the efficient regeneration of the human endometrium, which is preferentially evolved in humans and menstruating upper-order primates.

**WIDER IMPLICATIONS:**

The unique physiological organization of the human endometrial glandular element, its relevance to stem cell activity and scarless endometrial regeneration will inform reproductive biologists and clinicians to direct their future research to determine disease-specific alterations in glandular anatomy in a variety of endometrial pathological conditions.

## Introduction

**Figure 1.**
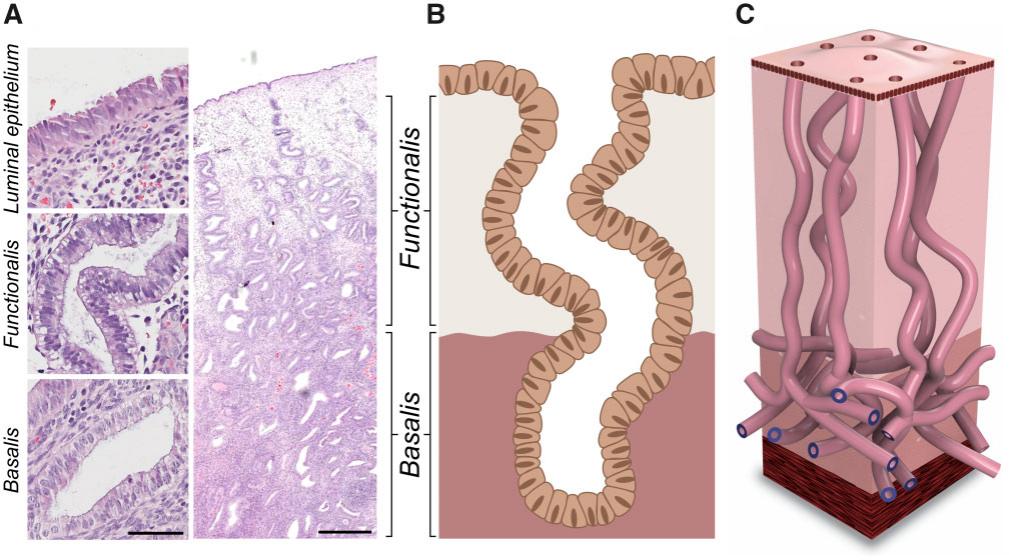
**Architecture of human endometrial glands: past and present**. (**A**) Haematoxylin and eosin stained human endometrial section at ×400 and ×20 magnification (scale bars = 50 and 500 μm, respectively). (**B**) 2D schematic of the pre-2020 consensus view of endometrial glandular architecture, with functionalis glands running a vertical course to the basalis glands and terminating in blind pouches. (**C**) 3D schematic of the novel endometrial gland arrangement based on recent findings, with basalis glands exhibiting a branching, mycelium-like configuration running perpendicular to functionalis glands.

The endometrium remains a poorly understood tissue in the human female reproductive tract, yet it plays the fundamental role of accepting and supporting an embryo and establishing pregnancy (Cha *et al.*, 2012). It is a dynamic and complex mucosal tissue that lines the uterine cavity and contains lumen and gland-forming epithelium, stroma, blood vessels and leucocytes (Lessey and Young, 2019). The human endometrium is organized into two functionally distinct areas; the superficial functionalis and the deeper basalis (Ferenczy and Bergeron, 1991; Gray *et al.*, 2001a) (Fig.  1A). Menstruation, or regular shedding of the whole superficial (functionalis) layer, is the most distinctive feature of human female reproduction. The human menstrual cycle, with its monthly remodeling, shedding and regeneration of the endometrium, is shared with only a few other species, including apes, Old World monkeys, four species of bats, the elephant shrew and spiny mice (Bellofiore *et al.*, 2017). However, it does not occur in any of the standard model organisms that undergo sexual reproduction, such as the mouse, zebrafish or fruit fly (Wang *et al.*, 2020). Although the multiple cellular subtypes of the human endometrium have been appreciated since the development of endometrial light microscopy, until recently the structural organization of the endometrial glands has been thought to be similar to that of the intestinal glands. Previous researchers examining human endometrium in the context of 2D tissue sections proposed the endometrial glands assume a single tubular architecture, terminating at the endometrial–myometrial junction, with a blunt end (Gargett *et al.*, 2016) (Fig.  1B). Recently, the novel 3D histological reconstruction of full-thickness, human adult endometrium, has demonstrated a much more complex architectural organization than previously thought (Tempest *et al.*, 2020) (Fig.  1C). These initial findings have been reconfirmed recently using tissue clearing-based methods (Yamaguchi *et al.*, 2021). The novel findings described by these two papers may provide plausible explanations for previously unanswered queries regarding endometrial regeneration and endometrial pathologies. In light of this novel description of a previously unknown endometrial glandular arrangement, this review aims to re-evaluate the current theories related to endometrial regeneration, location of stem/progenitor cells and the pathogenesis of some chronic endometrial conditions.

### History of the human endometrial glandular organization

Human endometrial glands have been described and depicted in all standard textbooks of gynaecology and histopathology as single, tubular, blind-ended entities running a vertical course from the luminal epithelium (LE) and terminating in the basalis on the endometrial–myometrial junction (Nogales *et al.*, 1969; Ferenczy, 1976; Nogales-Ortiz *et al.*, 1978; Gisela Dallenbach-Hellweg and Dallenbach, 2010; Kurman, 2019). Figure  1B illustrates the schematics of this previously prevailed (pre-2020) hypothesis of the human endometrial glandular organization.

The heterogeneity of epithelial cell subtypes existing within human endometrial glands has been appreciated for many years and has recently been explicitly confirmed by single-cell transcriptomic studies (Vento-Tormo *et al.*, 2018; Wang *et al.*, 2020). Both groups profiled different endometrial cell types, from non-pregnant human endometrium across the menstrual cycle (Wang *et al.*, 2020) and the first-trimester decidua (Vento-Tormo *et al.*, 2018). This approach allowed the identification of further discriminatory markers for endometrial cell subtypes, for example, Wang *et al.* (2020) reported ciliated epithelium to have a discrete transcriptomic signature compared with non-ciliated epithelium. Further, using known markers that distinguish the different cell subtypes (e.g. *LGR5:* leucine-rich repeat-containing G protein-coupled receptor 5), they also confirmed the existence of many other transcriptionally distinct, thus, proposed to be functionally different, endometrial epithelial cell sub-types (Wang *et al.*, 2020). Functional differences of human endometrial epithelial cells have also been suggested on the basis of their morphological or phenotypical characteristics or alluded to by their location in different sub-endometrial anatomical regions. For example, improved magnification and resolving power of microscopes facilitated the recognition of morphological differences, such as ciliated and non-ciliated cells in endometrial LE, with scanning electron microscopy (SEM) revealing further variances in endometrial epithelial subtypes (Motta and Andrews, 1976; Baraggino *et al.*, 1980; Nayel *et al.*, 1987). Future studies incorporating multiple methods will be able to provide explicit information on the distinct location, phenotype and function of endometrial epithelial subtypes.

#### Functionalis sub-region

Adult human endometrium is divided into two distinct anatomical regions; the upper functionalis, which contains glands loosely held together by supportive stroma, and the deeper basalis, consisting of branching glands and dense stroma (Ferenczy and Bergeron, 1991; Gray *et al.*, 2001a) (Fig.  1A).

The LE is a single layer of cuboidal epithelial cells lining the functionalis epithelium. It is supported by stromal fibroblasts (Evans *et al.*, 2014) and invaginates into the underlying cellular stroma to form tube-like functionalis glands. LE cells are less abundant than their glandular epithelial (GE) counterparts and, therefore, could be underrepresented in studies that used either whole endometrial tissue, or isolated epithelial cells (Evans *et al.*, 2014; Maclean *et al.*, 2020b). In the follicular phase of the cycle, the LE possess many ciliated cells (Nilsson *et al.*, 1980). Their role is to remove the secretions of the bordering cells and take an active part in the kinetics of the spermatozoa and in the capitation of the oocyte. The non-ciliated cells carry numerous, fairly long microvilli, with a greater number seen in the oestrogen-driven proliferative phase of the cycle, compared with the secretory phase (Baraggino *et al.*, 1980). The non-ciliated cells have apocrine secretory activity, are irregularly distributed and increase in number during the window of implantation (Baraggino *et al.*, 1980). The LE is the first maternal layer of cells that an embryo communicates with (Gray *et al.*, 2001a), and is thus central to determining the receptivity of the endometrium (Evans *et al.*, 2014).

When the ultrastructure of the GE is considered, there are mainly two types of cells; dark and clear cells (Demir *et al.*, 2002). Numerous cilia are seen on the apical pole of some clear cells, which is in contrast to the dark cells that lack cilia, nuclear channels and giant mitochondria profiles.

The functionalis epithelia proliferate massively in response to oestrogen in the proliferative phase and differentiate in response to progesterone, preparing to assist with conception by accepting the invading trophoblasts in the secretory phase (Lessey, 2013). The functionalis and basalis epithelial cells are morphologically similar during the proliferative phase, with a high nuclear-to-cytoplasmic ratio and specific features, such as elongated, allantoid shaped nuclei, which appear to be pseudostratified with dense chromatin and an inconspicuous nucleoli (Hendrickson, 2007).

During the early secretory phase, the GE cells accumulate glycogen particles, contain giant mitochondria and a nucleolar channel system. As the secretory phase proceeds, apocrine secretions and giant lysosomes become the most striking features (Cornillie *et al.*, 1985). The secretory phase glands have been described to be vertically orientated, perpendicular to the LE, with relatively large diameter lumens and irregular outer borders (Garry *et al.*, 2010). The decidual cells in the functionalis, and uterine natural killer (uNK) cells modulate the trophoblast function and endometrial angiogenesis via the secretion of growth factors, growth factor binding proteins, angiogenic factors and cytokines (Filant and Spencer, 2013; Lessey, 2013; Bulmer and Lash, 2019). The GE and their secretions play a fundamental role during early pregnancy by secreting substances that support blastocyst development (Lessey, 2013); its absence is associated with reduced conceptus survival (Gray *et al.*, 2001a,b, 2002). Deficient glandular activity during the secretory phase of the cycle in humans has been correlated with unexplained infertility (Dimitriadis *et al.*, 2006; Burton *et al.*, 2007). Furthermore, many studies have demonstrated the requirement of GE and their secretions for successful blastocyst implantation and stromal cell decidualization to support pregnancy in mice and humans (Filant and Spencer, 2013).

#### Basalis sub-region

The basalis, unlike the functionalis, is structurally stable, less proliferative and is the permanent compartment of the uterus, which persists throughout the lifespan of a woman and does not erode during menstruation or at parturition (Valentijn *et al.*, 2013; Kamal *et al.*, 2016). The basalis rests on the muscular subendometrial myometrium that contains denser stroma, and is less responsive to hormones compared to the functionalis; hence, it does not undergo rapid change throughout the menstrual cycle (Gray *et al.*, 2001a; Slayden and Keator, 2007; Lessey, 2013). With the advent of the essential role that stem/progenitor cells play in the regeneration and maintenance of an adult organ, the consensus was that the endometrial basalis is the location of endometrial stem cells (Prianishnikov, 1978; Gray *et al.*, 2001a; Gargett *et al.*, 2007; Valentijn *et al.*, 2013; Tempest *et al.*, 2018b).

Considering the architectural organization of the basalis glands, there were three descriptions pre-dating 2020 that suggested an alternative arrangement to the model illustrated in Fig.  1B. The first study, published in 2003, utilized multiphoton microscopy and concluded that endometrial glands appear to have both simple and branched tubular shapes (Manconi *et al.*, 2003). The focus of this paper was the architecture of the endometrial microvasculature, and the glands were only mentioned as an ancillary point, with an image that is difficult to interpret (Manconi *et al.*, 2003). Although subsequent 3D studies were reported by the same group, they did not further explore the glandular architecture (Simbar *et al.*, 2004; Manconi *et al.*, 2011).

The second study, published in 2010, postulated that single tubular basalis glands run a horizontal course parallel with the luminal surface for large parts of their length. However, the lack of robust data supporting this claim, which was based on the observation of 2D tissue sections, precluded the conclusions drawn (Garry *et al.*, 2010).

The third study, published in 2016, used whole-mount immunofluorescence and confocal imaging to visualize and characterize changes in the human endometrium. Three-dimensional renderings of optical sections of endometrial glands revealed increased glandular complexity of the human uterus compared with mice (Arora *et al.*, 2016). However, the study did not fully analyse or appreciate the detailed organization of human endometrium or its region-specific arrangement.

Although these early studies touched upon the possibility of an alternative glandular arrangement to the widely held consensus that their architecture is analogous to intestinal crypts, they did not fully appreciate or explore the magnitude of the complex glandular arrangement of the human endometrial glands.

### Recent discoveries in human endometrium

Over the last 2 years, two manuscripts have challenged and conclusively replaced the previously held hypothesis regarding the human endometrial epithelial architecture. Both studies, using two distinct but complementary methods, updated the preceding literature and concluded, in agreement, that the previously proposed single tubular glandular architecture was limited only to the endometrial functionalis glands. Furthermore, glands did not terminate in blunt ends in the basalis but in a complex, mycelium-like horizontal and intricate glandular arrangement, which occupied the deeper basalis layer of the human endometrium (Tempest *et al.*, 2020; Yamaguchi *et al.*, 2021).

The first study, published by our group in 2020, utilized histological 3D reconstruction with 100 consecutive paraffin-embedded 4 µM thick tissue sections, stained and aligned prior to generating 3D models with FreeD16 240 software (Tempest *et al.*, 2020), a method previously utilized in colon and breast tissue (Booth *et al.*, 2015; Baker *et al.*, 2019). Non-branching vertical functionalis glands and branching intricate basalis networks running horizontally to the myometrium were discovered for the first time, revolutionizing our knowledge of the human endometrial basalis architecture. We proposed that this ‘mycelium-like’ organization will have a functional advantage over the crypt configuration in terms of the conservation of progenitor/stem cells and regeneration (Tempest *et al.*, 2020). If the stem cells located in the basalis glands of a particular area are injured and lost following insults, their replenishment will be rapid owing to the complex basalis glandular configuration. Self-renewal of stem cells located in the adjacent basalis glands and their horizontal spread will ensure efficient restitution of the stem cells, regeneration of the basalis glands, with restoration of the entire endometrial glandular element at the deficient region. Conversely, a crypt-like conformation would leave the glands susceptible to complete denudation, owing to the loss of stem cell-rich basalis, with no means of replacement, resulting in areas of endometrial cavity lacking in glands at various life points such as following parturition or routine iatrogenic curettage. We also demonstrated that although the postmenopausal (PM) endometrium contained considerably fewer glands, they displayed a similar branching pattern to the basalis pre-menopausal glands (Tempest *et al.*, 2020).

The novel architectural arrangement of endometrial glands was confirmed in 2021 by Yamaguchi *et al.*, using the updated CUBIC tissue clearing protocol along with confocal and light-sheet fluorescence microscopy to reconstitute 3D views of the tissues (Yamaguchi *et al.*, 2021). Immunostaining with fluorescently labelled anti-cytokeratin 7 (CK7) antibody highlighted the endometrial gland structures. The manuscript also discussed cystically dilated, blind-ending occluded glands that were in a greater abundance in the secretory versus the proliferative phase of the cycle. These occluded cystic glands were detected regardless of age or menstrual cycle phase suggesting that they are basic components of the normal human endometrium. However, the number of the cystic glands present in the endometrium appears to be dependent on the hormonal milieu, with a high proportion seen in atrophic PM endometrium (McCluggage, 2006) and the endometrium exposed to progesterone receptor modulators (PRMs) (Ioffe *et al.*, 2009). In agreement with our description of mycelium-like basalis, they defined the plexus of the stratum basalis as the ‘rhizome’ owing to the similarity between the plexus and the rhizome in terms of morphology and function (Yamaguchi *et al.*, 2021).

### Implications for the epithelial stem cell niche

The concept of primitive (embryonic-type) cell(s) contributing to endometrial regeneration has been appreciated for nearly 100 years (Papanicolaou, 1933). Papanicolaou (1933) and Prianishnikov (1978) proposed that endometrial adult stem cells reside in the deep basalis layer, and their differentiation to be marked by functional changes (acquiring) in hormonal receptivity. This hypothesis, proposing that the human endometrium regenerates from the deeper basalis layer (which is the proposed germinal compartment that persists after menstruation and is responsible for the regeneration of the new superficial functionalis layer), remains to be the general consensus (Chan *et al.*, 2004; Valentijn *et al.*, 2013; Fayazi *et al.*, 2016; Gargett *et al.*, 2016; Theophilou *et al.*, 2018; Tempest *et al.*, 2018b). The endometrial stromal stem/progenitor cells contributing to human endometrial regeneration has been extensively studied and reviewed elsewhere (Darzi *et al.*, 2016; Gargett *et al.*, 2016; Tempest *et al.*, 2018b). Surface markers have been used to differentiate the basalis (stem/progenitor) epithelial cells from the functionalis, namely nuclear β-catenin, nuclear SRY-box 9 (SOX9), surface stage specific embryonic antigen-1 (SSEA-1) (Valentijn *et al.*, 2013) and surface marker N-cadherin (Nguyen *et al.*, 2017). Both nuclear β-catenin and nuclear SOX9 suggest activation of the Wnt signalling pathway in these cells, and this is an important pathway for adenogenesis in the endometrium and the intestine (Valentijn *et al.*, 2013).

The clonality of the human endometrial glands was initially assessed by two groups (Tanaka *et al.*, 2003; Kim *et al.*, 2005). The first paper, published in 2003, isolated individual endometrial glands from the stroma for the assessment of X chromosome inactivation patterns and to determine the clonal constitution of glandular cells, and the LE, in order to discuss the presence of endometrial stem cells (Tanaka *et al.*, 2003). The authors concluded that the cellular composition of individual glands was monoclonal and glands within a 1 mm^2^ area share clonality, concluding that the monoclonal composition of endometrial glands is regionally defined (Tanaka *et al.*, 2003). The authors argued that monoclonal growth is a hallmark of cancer cells and polyclonal growth is typical for benign tissue. Since their work demonstrated normal endometrial epithelial cells to exhibit a monoclonal origin, the authors proposed that endometrial glands must be an exception to the above theory, suggesting that possibly, owing to its iterative regeneration, the endometrial cells must be capable of long-term self-maintenance, with the properties of stem cells (Tanaka *et al.*, 2003). The authors postulated that stem cells with the same clonality are located in the basal layers in each gland, and, therefore, when endometrial cancer develops, this would arise from the stem cells at the base of the glands. The main drawback of this work is that the method used to extract the endometrial glands assumes that they are single blind-ending tubules akin to the intestinal glands. With the newly established endometrial glandular architecture, it is clear that this previous study would not have included the entire basalis glandular element but the superficial functionalis glands in their clonality assessment.

In the second clonality study published in 2005, Kim *et al.* tracked methylation patterns to assess stem cell progeny (Kim *et al.*, 2005). They employed similar glandular isolation protocols developed for the colon (Potten *et al.*, 1992), thus, for the aforementioned reasons, their methodology also was unlikely to have isolated all portions of the endometrial epithelial fraction but only examined the functionalis glandular portion. The authors hypothesized that methylation patterns in the endometrium increase from menarche to menopause, with relatively stable levels thereafter, assuming that the number of epigenetic marks is a reflection of the mitotic activity of endometrial stem/progenitor cells (Kim *et al.*, 2005). Different methylation patterns were found within the same uterus, consistent with individual glands being maintained by distinct stem cell niches that evolved independently (Kim *et al.*, 2005). Mathematical predictive analysis indicated that individual glands contained stem cell niches occupied by several long-lived stem cells. Symmetric and asymmetric cell divisions occurred in a stochastic manner, maintaining a constant number of adult stem cells in the endometrial gland niche (Kim *et al.*, 2005) and disputed the previously proposed monoclonality concept (Kim *et al.*, 2005). Their method, however, remained unsuitable to draw conclusions on clonality or stem cell arrangement of the entire epithelium spanning the full-thickness of the human endometrium.

**Figure 2.**
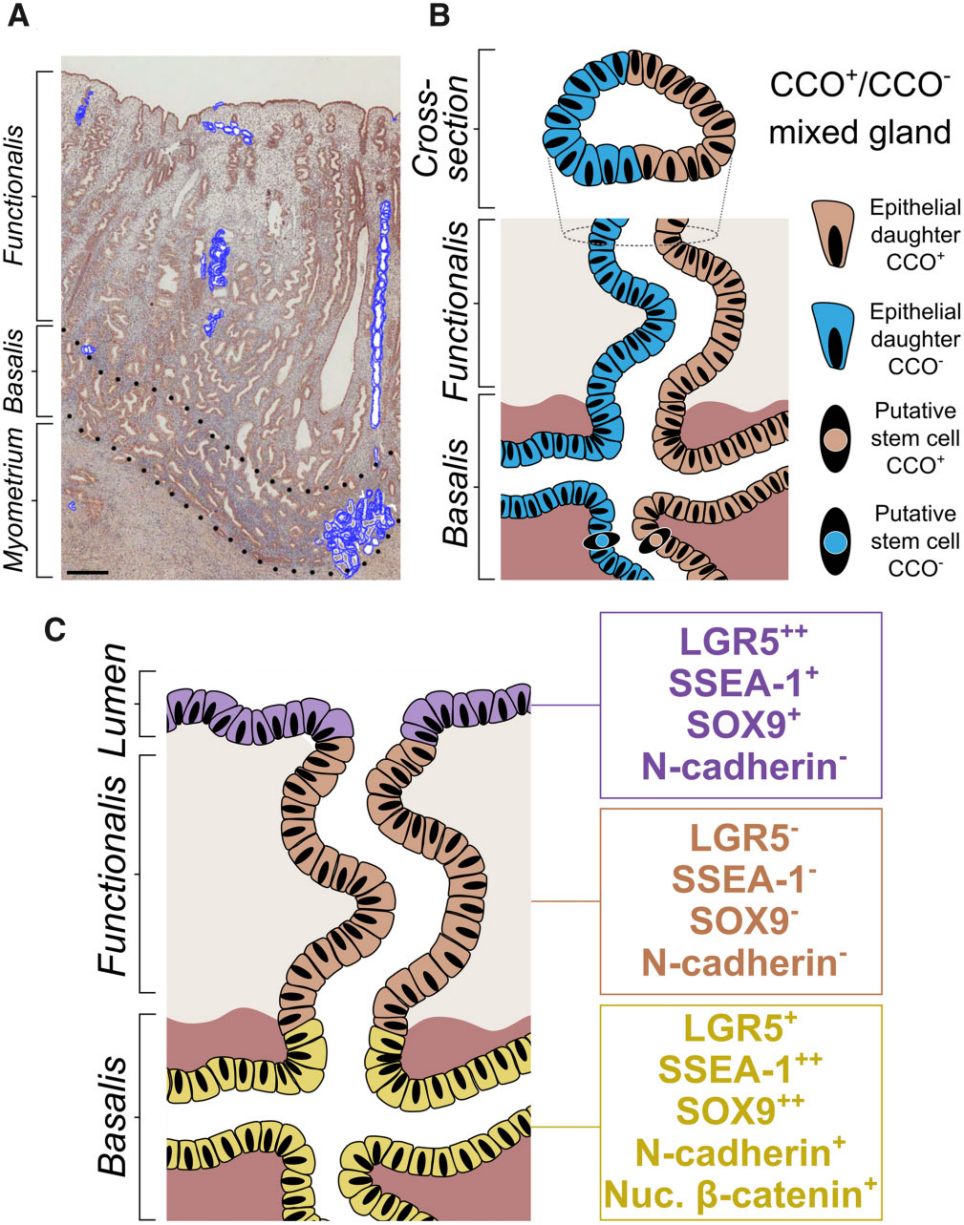
**Endometrial epithelial stem cell niche**. (**A**) Chromogenic immunostaining of cytochrome C oxidase (CCO) in the human endometrium highlighting the extent of CCO-deficient clonal expansion (blue glands). ×20 magnification (scale bar = 500 μm). (**B**) 2D rendering of the hypothetical origin of partially CCO-deficient functionalis glands arising from basalis-resident stem cells. Adapted from Tempest *et al.* (2020). (**C**) Putative stem cell niches in the endometrial lumen (leucine-rich repeat-containing G protein-coupled receptor 5 (*LGR5*^++^), stage-specific embryonic-antigen 1 (SSEA-1^+^), SRY-box 9 (SOX9^+^) and basalis (*LGR5^+^*, SSEA-1^++^, SOX9^++^, N-cadherin^+^, nuclear β-catenin^+^).

The recent discovery of a unique endometrial glandular organization, which is significantly different to the intestinal glandular architecture, highlighted deficiencies in the above two studies and necessitated re-evaluating clonality in the endometrial epithelium. In this pursuit, we used a method first adapted to humans by Taylor *et al.* (2003b) and later generalized for the identification of human epithelial stem cell niches in solid organs by Fellous *et al.* (2009) to confirm the existence of an endometrial epithelial stem cell that is long-lived and able to generate clonal expansion. *In vivo* lineage tracing using non-pathogenic mitochondrial DNA (mtDNA) mutations as clonal markers is a natural experiment that exploits spontaneously occurring mutations in the cytochrome C oxidase (CCO) (Wright, 2012) gene in long-lived stem cells. These mutations are inherited by daughter cells to produce a CCO-deficient clonal patch, thus allowing the existence and niche of stem cells to be identified. The observation of partially mutated endometrial glands combined with 3D models of CCO-labelled glands revealed clonal patches to contain a variety of cells expressing different markers for epithelial sub-regions, suggesting the existence of multipotent endometrial epithelial stem cells (Tempest *et al.*, 2020) (Fig.  2A and B).

The last two decades saw many researchers advancing our understanding of endometrial stem/progenitor cells that are responsible for the massive endometrial regeneration observed after menstruation and parturition. Many endometrial cell types have been presented for their *in vitro* stem cell functional activity, expression of markers described in other well-studied tissue stem cells, or owing to their specific location (Chan *et al.*, 2004; Gargett *et al.*, 2007, 2016; Gotte *et al.*, 2008; Masuda *et al.*, 2012; Valentijn *et al.*, 2013; Masuda *et al.*, 2015; Nguyen *et al.*, 2017; Tempest *et al.*, 2018b). It is widely believed that adult tissue-specific stem cells reside within a specialized microenvironment known as the niche, and stem cell phenotype/behaviour is regulated and maintained there (Spradling *et al.*, 2001). The niche senses the need for tissue replacement and compromises the adult stem cells, surrounding niche cell(s) and extracellular matrix. It is also a protective environment for the stem cell to maintain its genetic fidelity (Gargett and Masuda, 2010).

Further functions include the balance of stem-cell replacement (self-renewal) and provision of differentiated cells that are required for organ function (Gargett and Masuda, 2010). The stem cells receive a unique combination of extracellular signals directing them to maintain their ‘stemness’, and preserving their stem cell characteristics (Fuchs and Horsley, 2011; Nemeth and Karpati, 2014). The non-stem-cell residents of the niche must also be responsive to the needs of the organism/tissue, therefore, they can integrate external cues and mobilize the stem cells to exit the niche during homeostatic replacement (tissue maintenance) or upon injury (Fuchs and Horsley, 2011). The general consensus is that the basalis glands are the location of the human endometrial stem cell niche.

**Figure 3.**
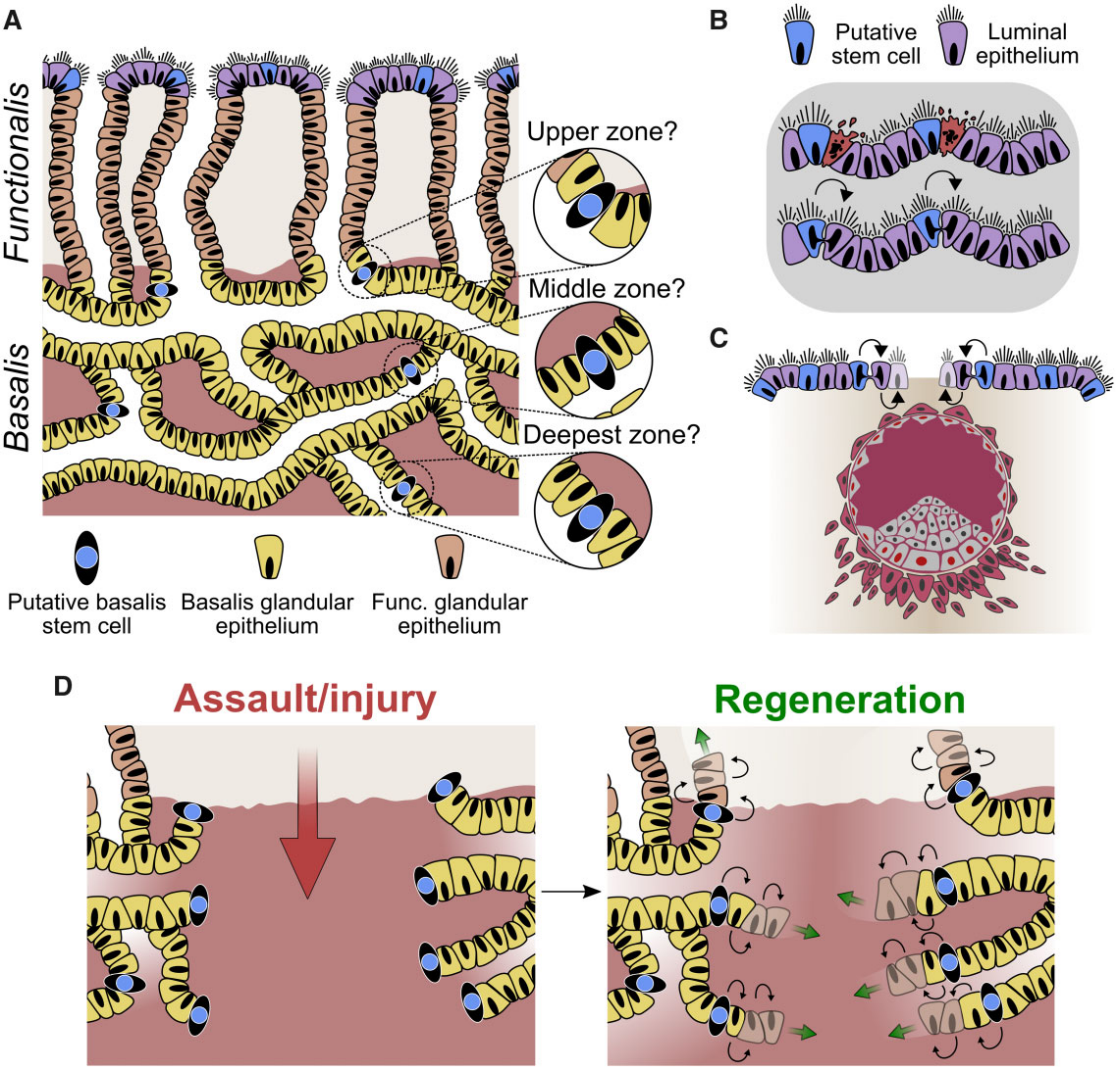
**Proposed endometrial epithelial regeneration**. (**A**) Schematic of the full-thickness endometrium highlighting the potential locations of epithelial stem/progenitor cells within the different zones of the basalis. (**B**) Maintenance of the luminal epithelium by resident stem/progenitor cells following daily shedding, desquamation events and (**C**) blastocyst implantation. (**D**) Model of epithelial regeneration via progenitors following complete destruction of the superficial and basalis layers of the endometrium. The mycelium-like configuration of the stem cell rich basalis will allow rapid replenishment of the lost basalis glands by horizontal spread in a way that would not be possible with a blind singular glandular conformation.

The first description of a basalis resident epithelial stem/progenitor population with *in vitro* adenogenetic properties described SSEA1, nuclear SOX9 and nuclear β-catenin to be expressed by basalis glands (Valentijn *et al.*, 2013). N-cadherin expressing epithelial cells also have been described to take a basalis glandular location and demonstrate stem/progenitor activity in an *in vitro* assay (Nguyen *et al.*, 2017) (Fig.  2C). The proposal that the cellular organization of the endometrial glands is hierarchical, in that the most primitive cells would be located in the very base of the basal glands (Gargett *et al.*, 2016), requires further scrutiny in the context of the mycelium-like organization of the endometrial basalis glands. If we draw parallels from the intestinal glands, the most primitive (reserve) stem cells are located at the ‘+4 position’, whereas the intestinal stem cells that regularly proliferate and participate in normal epithelial self-renewal and repair are situated in the base of the glands (Snippert *et al.*, 2010; Clevers, 2013). The more primitive ‘+4 position’ (thus more superficially placed) intestinal stem cells usually remain quiescent unless tissue repair secondary to massive injury is required (Snippert *et al.*, 2010; Clevers, 2013). Both studies depicting endometrial 3D architecture demonstrated sprouting of vertical functionalis glands from superficial and deeper levels of the horizontal basalis glands (Tempest *et al.*, 2020; Yamaguchi *et al.*, 2021). Therefore, the location of the most primitive and more active stem cell niche within this complex anatomy requires further study, and assumption based purely on location is not justified (Fig.  3A).

Out of the studies exploring the expression of stem cell markers, we would like to highlight LGR5, a proposed universal epithelial stem cell marker, since its expression pattern, demonstrating significantly higher *LGR5* expression in the LE compared with all other epithelial compartments, has elicited a novel hypothesis of the existence of a second stem cell niche in the endometrium (Tempest *et al.*, 2018a) (Fig.  2C). The knowledge of the endometrial glandular architecture in the context of the proposed LE-associated stem cell niche now compels researchers to examine the involvement of the LE in endometrial re-epithelialization/regeneration. The dual stem cell niche theory can be justified considering the endometrial requirements of daily maintenance and monthly regeneration of the whole functionalis (Fig.  3B).

## Implications for endometrial regeneration

### Menstruation

Menstruation is a remarkable process that is fundamental to human reproduction and gynaecology, and is limited to a few mammalian species. Endometrium is the primary target organ for ovarian hormones (Hapangama *et al.*, 2015), and the superficial LE and stratum functionalis are regenerated during the early proliferative phase. This phase is followed by further growth under the influence of rising oestradiol in the mid/late proliferative phase, differentiation and changes in cellular morphology in the secretory phase owing to the increase in progesterone, and either embryo implantation if the released oocyte is fertilized, or menstruation at the end of an infertile cycle (Maybin and Critchley, 2015; Hapangama and Bulmer, 2016). The recent updated architectural morphology of human endometrial glands may has implications for understanding the process of menstruation. The complex network of branched basalis glands allows us to appreciate how scarless regeneration can occur on a monthly basis following menstrual shedding. The mycelium/rhizome-like basalis glandular configuration will allow efficient regeneration similar to rhizomatous plants, such as grasses, that are able to regenerate following erosion. This ability, echoing the endometrial configuration, will ensure the conservation of progenitor/stem cells and safeguard regeneration longevity. Relying on a single blind-ending tube enriched with stem cells for the regular endometrial regeneration may allow inefficient regeneration, since the dedicated stem cells in the glandular bases can be easily lost or damaged. The horizontally inter-connecting, mycelium-like network configuration of the basalis will aid the self-preserving, self-renewing, scarless regeneration of the human endometrium by safeguarding the endometrial epithelial stem cell niche.

### Daily maintenance of endometrial integrity

As a consequence of external assaults, such as mechanical friction or infection, cells are continually lost and replaced from the surface of any epithelial tissue, including the skin and intestine; therefore, a similar daily cellular loss is likely to occur at the endometrial LE, which is exposed to the uterine cavity and external environment. The initial step of embryo attachment and invasion in the implantation process also occurs at the LE, and surface epithelium is rapidly replenished after this process. In cellular terms, LE exists at a relatively distant location from the basalis (up to 16 mm in the mid-secretory phase) (Fleischer *et al.*, 1999), therefore the daily maintenance and replacement after embryo invasion of the LE may require locally positioned cells with progenitor ability (Fig.  3B and C). Supporting this hypothesis, rapid LGR5^+^ epithelial cell proliferation can be observed in many other organs upon tissue damage (Ng *et al.*, 2014; Beumer and Clevers, 2016).

In our 2018 study, which examined LGR5 using *in situ* hybridization, LGR5 expressing epithelial cells were seen in the basalis, the well-established location of the endometrial stem cell niche (Valentijn *et al.*, 2013; Gargett *et al.*, 2016) but also in the LE (Tempest *et al.*, 2018a). When the LE is injured by iatrogenic procedures (e.g. endometrial curettage, endometrial scratching), the subsequent regeneration of the endometrial lumen and functionalis is rapid and complete, suggesting the existence of a stem/progenitor population in the LE.

We, therefore, hypothesize that it is possible for the human endometrium to have more than one epithelial stem/progenitor cell pool; one residing in the basalis (SSEA-1++, SOX9++ *LGR5*+, N-cadherin+, nuclear β-catenin+), supporting the massive regeneration of the functionalis after menstrual shedding or parturition; while the other (*LGR5*++ SSEA-1+ SOX9+, N-cadherin-) supports the embryo-implantation process, and maintains the LE cells that are likely to be lost on a daily basis (Fig.  2C). This hypothesis agrees with the SEM studies of human endometrium, the endometrial injury model of the rabbit (Ferenczy and Richart, 1974), and neo-natal endometrial glandular development in humans (Cooke *et al.*, 2013). The persistent expression of the progenitor cell markers SOX9 and SSEA-1 in the LE, with concomitant high *LGR5* expression, corroborate further the above hypothesis (Barker and Clevers, 2010; Valentijn *et al.*, 2013). Although *LGR5*-rich LE may demarcate a second endometrial epithelial stem cell niche (Tempest *et al.*, 2018a), in the work described in our 2020 paper we did not observe any CCO deficient patches spanning a large portion of the LE in the samples we examined. Therefore, a LE contribution to the functionalis glands regeneration could not be substantiated with the available evidence to date. The lack of large CCO deficient LE patches could be related to the high cellular turnover required for the LE progenitor pool, thus they are not sufficiently long-lived to accumulate CCO mutations. Further work is required to investigate the contribution of the LE to endometrial glandular regeneration and how LE is regenerated after menstrual shedding of the functionalis.

### Parturition

During pregnancy, the endometrium undergoes decidualization, a process that involves morphological and functional differentiation of human endometrial stromal cells, essential for establishing a healthy pregnancy (Coulam, 2016). Prior to and after parturition, massive regeneration takes place in the endometrium through a process of immune cell infiltration, re-epithelialization of the LE and proliferation. The process takes ∼2–3 weeks, and the endometrium is less active from Weeks 4 to 6 after parturition, appearing histologically as early proliferative phase endometrium (Sharman, 1953; Mulic-Lutvica *et al.*, 2001).

Given the extensive changes that occur in the postpartum endometrium, including shedding of the majority of the decidua and slow endometrial regeneration compared to that after more superficial injuries, we postulate that it is the basalis epithelial progenitor cells that are responsible for regeneration. Further, Huang *et al.* (2012) report that in the mouse the epithelial compartment of the endometrium retains its epithelial identity during postpartum regeneration. Considering the recently discovered glandular organization, we can propose that a plexus of glands in the basalis are retained post-parturition, similar to after menstruation, and it is from these that endometrial regeneration occurs. Recent mouse studies postulate that scarless postpartum endometrial regeneration requires sub-endometrial myometrial contractions to produce an essential transient hypoxic environment, which activates the stem cell niche (Yoshii *et al.*, 2014). If this observation is relevant to human postpartum endometrial regeneration, or if human endometrial stem cell activation in general is dependent on myometrial contractions/hypoxic environment, is yet to be determined. There is a paucity of experimental data on the cellular mechanisms that regulate human postpartum endometrium, which would be beneficial to further understand postpartum endometrial pathologies, including invasive placentation and caesarean scar niche.

### Regeneration after iatrogenic destruction

The endometrial ablative procedures, used to treat excessive menstruation, are designed to destroy the deeper endometrial layer, abolish stem cells that reside there and offer complete cessation of menstruation owing to lack of subsequent regeneration (Wortman, 2018). Amenorrohea, unfortunately, is not the case for numerous women; even following extensive iatrogenic destructive procedures (Tresserra *et al.*, 1999) the endometrium regrows (25–75% of women having endometrial ablation), and women will continue to bleed (Gimpelson, 2014; Muller, 2015). The newly discovered organization of the basalis glands could explain how, even if a small area of complex and intricate root-like basalis glandular configuration is retained, the entire glandular basalis element could be replenished via horizontal branching (Fig.  3D). Unlike the rapid LE regeneration, when more extensive destruction of the deeper basalis has occurred (e.g. with endometrial ablation, repeated curettage post pregnancy) the subsequent regeneration takes longer and may not be complete (e.g. Asherman’s syndrome). The basalis glands (protected from external influence) thus may be the location for the primary, slow proliferating stem-cell niche responsible for regenerating all epithelial regions. Identifying specific markers to isolate LE cells and basalis epithelium separately will allow functional studies using appropriate complex 3D *in vitro* models to further investigate the differential stem cell and adenogenic properties of cells originating from these two anatomical areas and to confirm the dual stem cell theory.

### Postmenopausal endometrium

In the absence of endogenous/exogenous hormones, the PM endometrium enters a non-proliferative, quiescent state, with a thin basalis-like endometrium and LE only. The healthy PM endometrium demonstrates higher androgen receptor expression than its proliferative, premenopausal counterparts (Kamal *et al.*, 2016), which we have previously hypothesized may contribute to its quiescent state (Maclean *et al.*, 2020a). Histology of the PM endometrium described by McCluggage *et al.* shows inactive glands contained in a compact stroma, similar in appearance to the stratum basalis in premenopausal endometrium (McCluggage, 2011). This similarity has been confirmed in 3D by recent works (Tempest *et al.*, 2020; Yamaguchi *et al.*, 2021). Interestingly, the atrophic PM endometrium is commonly found to have cystic dilation of some glands (Buckley and Fox, 2002). Endometrial glandular growth and proliferation requires oestrogen and can be induced in PM endometrium with the administration of exogenous steroids (Ekberg, 2014; Hapangama *et al.*, 2015). We propose that the deeply located stem cells in the PM endometrial basalis maintain the glands that grow upwards towards the LE. Severe lack of oestrogenic signal leads to growth arrest and may result in the frequently observed, blocked, cystically dilated PM endometrial glands. Iatrogenic hormonally induced PM endometrial regeneration is expected to occur from the basalis/lumen, but conclusive proof is currently absent as to this process, and further research is needed.

### Implications of endometrial changes reported with exogenous hormones

PRMs have been shown to be useful in many gynaecological conditions, including fibroids, endometriosis and as potential female contraceptives (Hapangama *et al.*, 2001; Hapangama, 2003). However, their long-term use has been hampered by concerns related to associated endometrial changes, including inactive endometrium with cystically dilated glands and epithelium with increased apoptosis on a background of compact non-decidualized stroma (Spitz, 2009; Williams *et al.*, 2012). The degree of progesterone receptor antagonistic activity had been proposed to be the cause for the differential histological changes detected between different PRMs (Donnez *et al.*, 2012). The oestrogenic effect on the endometrial epithelium is known to be pro-mitotic and induce glandular growth (Hapangama *et al.*, 2015; Valentijn *et al.*, 2015; Kamal *et al.*, 2016). Considering the findings by Yamaguchi *et al.* (2021), which revealed these cystic glands to be blocked functionalis glands arising from the basalis, we can postulate that the PRMs, with more agonistic action, may prevent the oestrogen-driven vertical growth of the functionalis glands to the LE, thus producing the typical inactive epithelial phenotype accompanied by cystically dilated glands.

## Implications for endometrial pathologies

### Adenomyosis

Adenomyosis is a benign uterine disease defined by the pathological location of endometrial epithelial glands and stroma in the myometrium, with surrounding muscular hypertrophy, manifesting clinically as abnormal uterine bleeding, pelvic pain and infertility (Ferenczy, 1998; McCluggage and Robboy, 2009; Garcia-Solares *et al.*, 2018). The aetiology and pathogenesis of adenomyosis are poorly understood. A number of human and experimental studies support the theory of invagination of the endometrial basalis into the myometrium through a process of tissue injury and repair (Leyendecker *et al.*, 2009; Leyendecker and Wildt, 2011; Vannuccini *et al.*, 2017; Yamaguchi *et al.*, 2021). However, other unproven theories exist, such as *de novo* development from metaplasia of displaced embryonic pluripotent Müllerian or epithelial remnants, and differentiation of adult endometrial stem cells within the myometrium (Chan *et al.*, 2004; Chen *et al.*, 2010).

**Figure 4.**
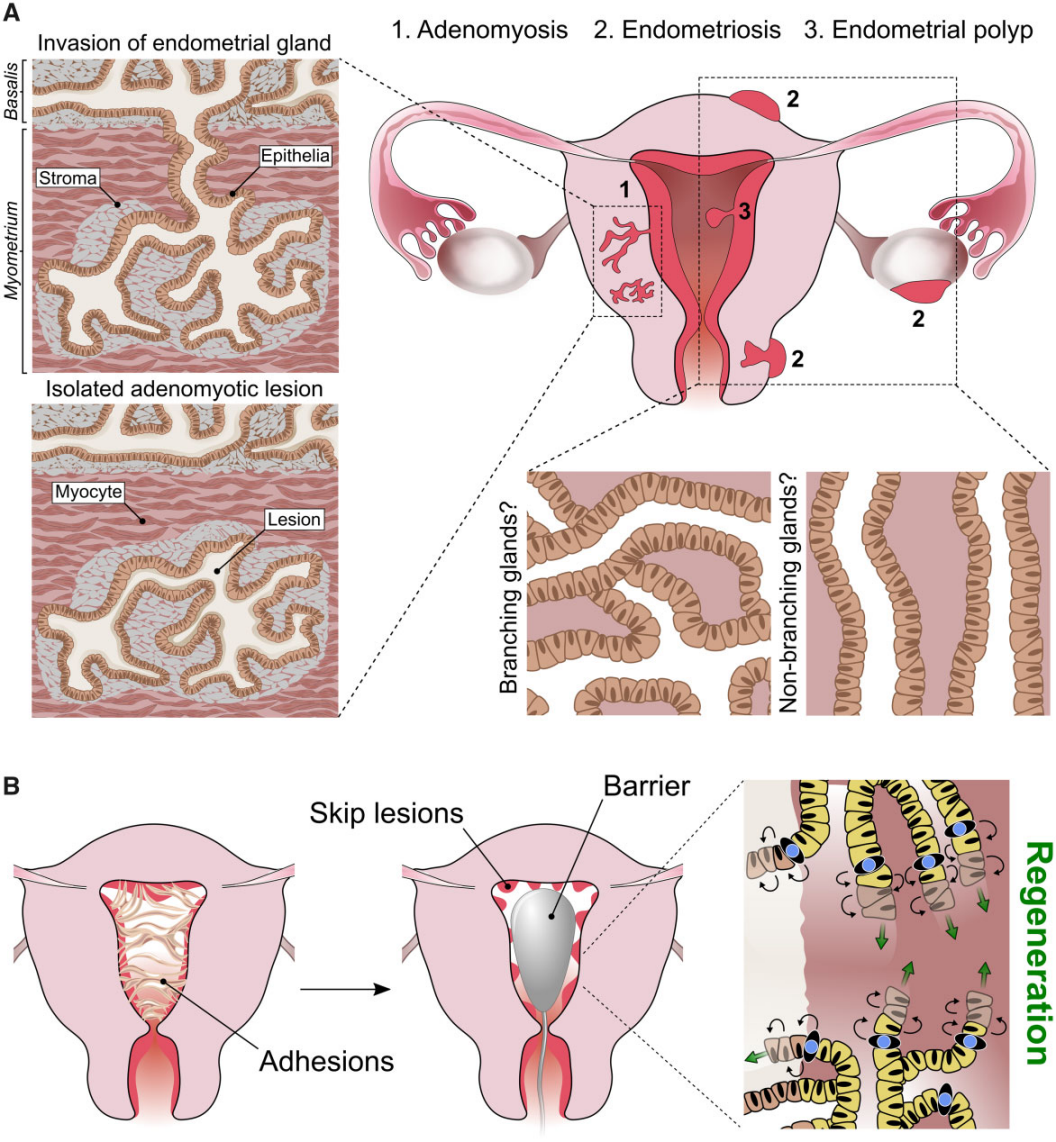
**Relevance of epithelial histoarchitecture to endometrial pathologies**. (**A**) Schematics show the ant colony-like structure of adenomyotic lesions containing epithelial glands with multiple branch points (Yamaguchi *et al.*, 2021). Lesions may be connected directly to the endometrial basalis, supporting the theory of eutopic glandular invasion of the myometrium, or isolated lesions, hinting at an alternative origin. The glandular architecture of endometrial polyps and endometriotic lesions (including superficial, ovarian and deep-infiltrating endometriosis) remain to be elucidated. They may have a complex-branching or non-branching glandular conformation. (**B**) Proposed model of endometrial regeneration in Asherman’s syndrome; following surgical removal of intrauterine adhesions and barrier insertion to inhibit recurrence, basalis networks from neighbouring skip lesions are able to expand parallel to the myometrium in a horizontal plane by means of resident stem/progenitor cells, subsequently restoring the whole glandular element.

There is a spectrum of histological definitions of adenomyosis, based on either the depth between the adenomyotic lesion and the deepest basalis endometrial gland (Levgur *et al.*, 2000; Sammour *et al.*, 2002), or by the proportion of myometrial involvement (Mark *et al.*, 1987; Ferenczy, 1998). However, a uniformly agreed consensus on the histopathological criteria required for a diagnosis is yet to be devised (Vercellini *et al.*, 1993; Seidman and Kjerulff, 1996). A number of MRI-based classification systems categorize adenomyosis as ‘intrinsic’ and ‘extrinsic’ based on the location of the adenomyotic foci within the myometrium (Mehasseb *et al.*, 2010; Kishi *et al.*, 2012, 2017), and this hints at different pathogenic processes. Yamaguchi *et al.* (2021) described the 3D appearance of adenomyotic lesions as an ‘ant-colony like’ structure of ectopic endometrial glands within the myometrium, suggesting direct invasion of eutopic endometrial glands into the myometrium and supporting the invagination theory of adenomyosis. However, whether these adenomyosis lesions are deep-sited endometrial basalis glands is not evident, and without further histological classification of adenomyotic lesions, visualization of a connecting gland between the basalis and the myometrium in itself does not provide proof of mucosal invasion of the myometrium (Fig.  4A).

The ‘ant-colony like’ adenomyotic lesions described by Yamaguchi *et al.* may represent ‘intrinsic’ adenomyosis. The CUBIC tissue clearing protocol and visualization techniques utilized in the manuscript (Yamaguchi *et al.*, 2021) could be applied to understand the histoarchitecture of other proposed subtypes of adenomyosis in the context of endometrial 3D configuration, to elucidate further the pathophysiological processes leading to their development. Recent murine studies have provided interesting experimental evidence that iatrogenic procedures causing either mechanical or thermal damage to the endometrial–myometrial interface may initiate adenomyosis (Hao *et al.*, 2020). We propose that this region in the uterine wall may be the inferior boundary of the endometrial epithelial stem cell niche, representing a barrier to contain stem cell activity, which may regulate and direct the growth of functionalis glands towards the endometrial lumen. Therefore, its disruption may cause disconcerted endometrial growth/regeneration towards the myometrium, instigating adenomyosis. However, future research is required to examine this hypothesis further.

### Endometriosis

Endometriosis is a highly prevalent chronic disease (Shafrir *et al.*, 2018) that significantly impacts the affected person’s life (Nnoaham *et al.*, 2011; NICE, 2017a,b) and is characterized by the extra-uterine existence of endometrium-like tissue (Burney and Giudice, 2012). Endometriosis lesions are grouped into three broad categories; superficial peritoneal, ovarian endometrioma and deep-infiltrating (Tosti *et al.*, 2015) and their pathogenesis is not yet fully understood (Sourial *et al.*, 2014; Hill *et al.* 2020). Cells originating from the stratum basalis are geographically protected from mechanical displacement during menstruation. However, in women with endometriosis, endometrial cells are postulated to be shed with menstruation (Hapangama *et al.*, 2019) and potentially implanted in the pelvis after retrograde menstruation, where they demonstrate a cyclical response to ovarian hormones and resist apoptosis (Hapangama *et al.*, 2008b,c, 2010) leading to a state of sustained proliferation (Hapangama *et al.*, 2009). The eutopic endometrium of women with endometriosis is postulated to be different to that of women without endometriosis. This had been linked to some of the associated symptoms, such as infertility (Macer and Taylor, 2012), and the reason for retrogradely transferred menstrual endometrium to be deposited and initiate ectopic lesions in only some women (Hapangama *et al.*, 2008a, 2012).

It now remains to be explored if the 3D organization of the eutopic endometrium of patients with endometriosis differs from those without endometriosis. Such findings could help us understand the as yet unproven pathophysiology of endometriosis and the multifaceted nature of the condition. Determining the 3D architecture of different endometriotic deposit subtypes may also shed light on their origin in the context of endometrial sub-regions and explain their pathogenesis and potential ability for propagation and responsiveness to different therapies (Fig.  4A).

### Endometrial polyps

Endometrial polyps are focal neoplasms that typically protrude into the uterine cavity above the adjacent surface epithelium (Nijkang *et al.*, 2019). They often present with abnormal uterine bleeding, and although the exact prevalence is unknown they remain the most frequently observed pathological finding in the uterus and are usually benign (Nijkang *et al.*, 2019). Sessile polyps have a flat implantation base, whereas pedunculated polyps are attached to the uterine surface with an elongated pedicle (Di Spiezio Sardo *et al.*, 2015). Macroscopically, endometrial polyps have a smooth and spherical or cylindrical appearance, ranging from a few millimetres to several centimetres in size (Vitale *et al.*, 2021).

Owing to their persistent nature, endometrial polyps are postulated to originate from the basalis and are expected to be less responsive to ovarian hormones. However, if any tissue from polyps is shed with menstruation is unknown, and available evidence regarding cyclical changes in the polyps compared with adjacent endometrium is controversial (Kasap *et al.*, 2016). Many reported features of endometrial polyps are consistent with those of the basalis. These include the expression of many angiogenic and stem cell markers, steroid receptors and the presence of leukocyte subtypes (Al-Jefout *et al.*, 2009; Tokyol *et al.*, 2009; Xuebing *et al.*, 2011). High microvascular density has also been reported in the basalis layer of the normal endometrium and in endometrial polyps (Makhija *et al.*, 2008; Tokyol *et al.*, 2009). Similar to the less dynamic endometrial basalis layer, which does not demonstrate decidualization, stroma of the endometrial polyps is also reported to be resistant to decidualization with reduced oestrogen receptor (ER) expression (Mittal *et al.*, 1996). However, unlike proliferatively quiescent basalis glands, the epithelium of endometrial polyps is highly proliferative (Miranda *et al.*, 2010) with a concomitant reduction in inhibitors of apoptosis (Taylor *et al.*, 2003a) yet a higher ER expression than the adjacent endometrium (El-Hamarneh *et al.*, 2013), which may contribute to higher hormone sensitivity and pathogenesis.

The recently described structure of the endometrial basal glands may have important implications for our current understanding of the pathogenesis of endometrial polyps (Tempest *et al.*, 2020; Yamaguchi *et al.*, 2021). By drawing parallels with the histological architecture of the epithelial component of the polyps and normal endometrial sub-regions or with endometrial hyperplasia, knowledge of the 3D microarchitecture of the endometrium may assist in predicting the natural life course of the polyp as well as their risk of recurrence or malignant transformation (Fig.  4A). Such knowledge will be invaluable to guide therapy and surveillance.

### Asherman’s syndrome

Asherman’s syndrome is a rare, acquired condition characterized by the formation of scar tissue within the uterine cavity and/or the cervix (Yu *et al.*, 2008). The condition typically develops in the wake of over-zealous curettage following parturition but can also be caused by endometrial ablation procedures (Yu *et al.*, 2008) and infective causes, including schistosomiasis and genital tuberculosis (Dreisler and Kjer, 2019). The incidence of Asherman’s syndrome varies between 2% and 22% in infertile patients (Singh *et al.*, 2014), but these figures vary depending on diagnostic criteria and geographic location (Gargett and Healy, 2011). Although this syndrome may be unrecognized in those who are not trying to conceive, classical symptoms include light menstrual bleeding, amenorrhoea, and infertility. Intrauterine adhesions develop secondary to trauma and loss of the basal endometrial stem cell niche (Gargett and Healy, 2011; Valentijn *et al.*, 2013; Tempest *et al.*, 2018b). Macroscopically, intrauterine adhesions vary in size and may be filmy or dense. They are commonly avascular (Deans and Abbott, 2010) and the basal and functional layer of the endometrium are indistinguishable; stroma is replaced by an epithelial monolayer that does not respond to hormonal stimulation (Foix *et al.*, 1966; Yu *et al.*, 2008).

When the uterine cavity is completely obliterated after iatrogenic injuries, small pockets of functional endometrium, known as ‘skip lesions’, that are preserved, may cause chronic pelvic pain and amenorrhoea (Amin *et al.*, 2015). The functional consequences and associated symptoms may reflect adenomyosis, and endometrium above areas of complete obstruction may be abnormally thin, although the specific pathology behind this process remains unexplained (Amin *et al.*, 2015). After surgical separation of intrauterine adhesions, intrauterine devices (IUD) or solid barriers, such as Foley catheters and balloon stents, are commonly inserted to form a physical barrier to prevent apposition of uterine walls and without that the risk of recurrence remains high (Pabuccu *et al.*, 1997; Conforti *et al.*, 2013; Huang *et al.*, 2020). We can postulate that by keeping the endometrial cavity open, the mycelium-like basalis glands from adjacent skip lesions will be allowed to repopulate the stem cell niche overlying the previously adherent myometrium (Fig.  4B). Inert devices are the recommended post-surgical intrauterine barrier type, while the use of Copper or progestin containing IUD is not recommended (Surgery, 2017). The negative effects of progestin on endometrial growth (Valentijn *et al.*, 2015) is well established, with no clinical data supporting progestin-IUD use in Asherman’s syndrome, while some authors advocate the post-surgical use of copper IUD for intrauterine adhesions (Vesce *et al.*, 2000). The copper-IUD has previously been proposed to provoke a local inflammatory response (Patai *et al.*, 1989). However, gene expression studies have demonstrated significant alterations in genes regulating immune and inflammatory pathways with the progestin-IUD, while the Copper-IUD showed no effect (Smith-McCune *et al.*, 2020). Inflammation may either support or inhibit stem cell proliferation, depending on the severity and chronicity of the immune response (Kizil *et al.*, 2015), which may explain the potential benefit of copper-IUD in comparison with progestin-IUD. Furthermore, we postulate that the recommended large inert devices are likely to elicit minimal but optimum amount of inflammation, providing a large, most advantageous free myometrial surface, on which the stem cells in basalis glands can propagate horizontally, restoring the epithelial niche, with subsequent regeneration of the entire endometrial glandular element (Fig.  4B).

### Placenta accreta, increta and percreta

Placenta accreta, increta and percreta occur when the chorionic villi pathologically invade the decidual basalis, myometrium or perimetrium, respectively, and are collectively termed placenta accreta spectrum (PAS) disorders (Silver and Branch, 2018; Jauniaux *et al.*, 2019). PAS disorders are associated with significant maternal morbidity and are potentially life-threatening complications of pregnancy (Jauniaux *et al.*, 2018; Silver and Branch, 2018). It has been posited that caesarean sections and other uterine surgeries, including endometrial ablation, curettage and myomectomy, enhances the risk of PAS through damage to the endometrial basalis layer and/or the hypoxic environment created by scar tissue (Jauniaux *et al.*, 2018). Interestingly, it has been proposed that uterine contraction evokes ischemia and local hypoxia, generating a special microenvironment that promotes scarless healing in the endometrium (Yoshii *et al.*, 2014). The damaged myometrium, for example, after uterine surgery, may not be able to produce scrupulous myometrial contractility to ensure local hypoxia at the complex basalis resulting in endometrial scarring and consequential deficient endometrium. An increased incidence of PAS disorders is also associated with Asherman’s syndrome (Roy *et al.*, 2010).

The newfound endometrial microarchitecture has important connotations for the pathophysiology of PAS disorders. The risk of PAS is high when the placenta is situated in the lower segment, particularly after a previous caesarean section. The likelihood of PAS disorders increases with the number of previous caesarean sections (Silver and Branch, 2018; Jansen *et al.*, 2020). It has been postulated that the destruction of the basalis may represent a deficiency in the formation of the spongiosus layer of the decidua, thus facilitating abnormal placentation (Shepherd and Mahdy, 2021). The horizontal basalis glands are highly interconnected and form multiple branch points from which the functionalis glands originate (Tempest *et al.*, 2020; Yamaguchi *et al.*, 2021). The precise extent to which these structures are interconnected throughout the endometrial cavity, particularly in the lower segment of the uterus, where the caesarean section incisions are usually placed, remains unknown. The basalis glandular configuration differences may exist in different regions of the uterine cavity, which may alter the impact of operative surgery on endometrial basalis structure and function; this requires urgent investigation. Examination of the endometrial basalis glandular organization in the vicinity of the caesarean section scar may provide information on the pathogenesis of conditions associated with imperfect repair and regeneration of the uterine wall after caesarean scar, such as PAS conditions and the recently described caesarean section niche disorder (Al Mutairi and Alrumaih, 2020).

### Subfertility, recurrent pregnancy loss and recurrent implantation failure

Subfertility and infertility are common conditions, defined as a delay in conceiving, and the inability to conceive after 12 months of regular unprotected intercourse, respectively (Thurston *et al.*, 2019). Subfertility affects up to one in seven couples (NICE, 2017a,b). Multiple factors and pathologies have been found to be associated with subfertility/infertility, including advanced maternal age, ovulatory disorders, endometriosis, tubal disease, uterine abnormalities and male factor infertility (NICE, 2017a,b). Despite this, up to 30% of all cases are thought to be unexplained (Farquhar *et al.*, 2019), and endometrial aberrations may explain at least some of these.

Recurrent pregnancy loss (RPL) (Eshre Special Interest Group *et al.*, 2018) is one of the major challenges for clinicians; ∼50% of cases remain unexplained in aetiology, and the management options are limited and poorly understood (RCOG, 2011; El Hachem *et al.*, 2017). Many studies have now suggested an association between unexplained RPL and a defective endometrium (Quenby *et al.*, 2007; Hapangama *et al.*, 2008a; Lucas *et al.*, 2016; Ticconi *et al.*, 2019; Dimitriadis *et al.*, 2020; Tempest *et al.*, 2021). Additionally, inadequate endometrial receptivity may be responsible for two-thirds of recurrent implantation failure (RIF), in which there is failure to implant after multiple transfers of good quality embryos using ART (Hapangama *et al.*, 2008a; Simon and Laufer, 2012; Bashiri *et al.*, 2018; Tempest *et al.*, 2021). These devastating conditions are important reproductive health issues with many unanswered questions, leading to significant emotional and psychological impact for patients and their partners (De Klerk *et al.*, 2007; Koert *et al.*, 2019; Voss *et al.*, 2020).

The intricacies of human embryo implantation are still not fully understood. Owing to both technical and ethical limitations, it is not possible to study human embryo implantation *in vivo.* Current knowledge about implantation has mainly been obtained from animal models and *in vitro* 2D monolayer culture systems, which fail to represent the 3D architecture and physiology of the human process (Cha *et al.*, 2012; Stern-Tal *et al.*, 2020). Recent data revealing the novel 3D architecture of the endometrial epithelial compartment has now shed light on possible new explanations for the pathogenesis of many poorly understood endometrium-related conditions, including RPL and RIF (Tempest *et al.*, 2020; Yamaguchi *et al.*, 2021). Within mice, 3D visualization now confirms that crypts formed from the invagination of the LE have direct communication with existing glands (Yuan *et al.*, 2018). Glandular secretions can therefore be directly delivered to the blastocysts at the site of implantation within these crypts (Yuan *et al.*, 2018). Deletion of the planar cell polarity gene *Vlang2* is seen to result in an inferior shallow crypt structure and underdeveloped non-extending glands, subsequently leading to poor pregnancy outcomes (Yuan *et al.*, 2016). Whilst this provides further evidence for the importance of glandular topography in embryo implantation, it is important to note that non-menstruating and non-regenerating endometrial mouse models are limited when considering implantation within humans (Yuan *et al.*, 2016; Tempest *et al.*, 2020).

The described endometrial aberrations associated with all of these conditions of recurrent pregnancy failure are persistent in the functionalis layer of the endometrium, where embryo implantation occurs. The functionalis layer is shed with each menses, therefore, the cause for the lasting endometrial functionalis defects should originate from the basalis, which is responsible for the repetitive regeneration of the functionalis.

We can speculate that patients who suffer from infertility, RPL and RIF may have an underdeveloped or abnormal network of basalis glands and therefore stem cell population. The abnormal stem cell population would ultimately lead to a defective regeneration of the functional endometrium and LE, affecting the key components for successful implantation. We consider the 3D investigation of the endometrial epithelium and embryo implantation within humans to be an important avenue for future research (Tempest *et al.*, 2020; Yamaguchi *et al.*, 2021).

## Future directions

The newly revealed human endometrial glandular organization has provided explanations for long-standing queries regarding the efficient, frequent and scarless regeneration of the endometrium. However, pertinent questions remain unanswered: the precise characteristics of the stem/progenitor cell(s) of the endometrium that can repopulate all epithelial subpopulations each month; their exact location within the branching basalis glands; how their function is orchestrated throughout the cycle and during pregnancy establishment; how they are replenished after massive assault/injury and their contribution to LE regeneration; and if the microarchitecture of the endometrium differs across the uterine cavity. Most models of reproductive pathologies are based on non-menstruating species, such as mice, which are not directly translatable to humans. For example, the murine endometrium does not contain horizontal basalis glands but vertical crypt-like structures. Therefore, it is important to consider these fundamental differences when designing research studies to expand the current understanding of endometrial and menstrual pathologies. In this context, *in vitro* 3D culture models using patient-derived primary cells will provide an important and clinically transferable opportunity to study human endometrial conditions, including embryo-implantation and fertility-related research. Indeed, recent advances in the generation of physio-mimetic, hormone-responsive endometrial organoids (Turco *et al.*, 2017; Alzamil *et al.*, 2021) will significantly enhance co-culture systems via the incorporation of gland-like structures. Such structures, and the cell types they contain, could be tailored to mimic the glandular arrangement of the endometrium. Current 3D models, however, are insufficiently sophisticated in terms of architectural complexity and inclusion of all different cellular subtypes of the human endometrium. The technological advances in biomaterials, mesofluidic devices, organ-on-chip platforms and knowledge generated from single-cell transcriptomics will allow future development of suitably complex and directly clinically translatable 3D human endometrial *in vitro* models.

## Conclusion

The recently discovered unique 3D architecture of the human endometrial epithelium has answered some enduring questions about efficient and scarless endometrial regeneration in women while posing many further questions related to the micro-architectural arrangement of the endometrium in health and diseases. Future research focus should be directed to develop clinically translatable 3D human endometrial *in vitro* models that are accurately mimicking the architectural complexity of the human endometrium, including all different cellular subtypes, to improve our current understanding of endometrial pathological conditions to aid development of curative treatments.

## Data availability

The data underlying this article are available in the referenced works.
